# Genomics and Functional Genomics of Alzheimer’s Disease

**DOI:** 10.1007/s13311-021-01152-0

**Published:** 2021-12-21

**Authors:** M. Ilyas Kamboh

**Affiliations:** Department of Human Genetics, Graduate School of Public Health, University of Pittsburgh, Pittsburgh, PA USA

**Keywords:** Alzheimer’s disease, SNPs, Genomics, Genome-wide association studies, Functional genomics

## Abstract

Alzheimer’s disease (AD) is a complex and multifactorial neurodegenerative disease. Due to its long clinical course and lack of an effective treatment, AD has become a major public health problem in the USA and worldwide. Due to variation in age-at-onset, AD is classified into early-onset (< 60 years) and late-onset (≥ 60 years) forms with early-onset accounting for only 5–10% of all cases. With the exception of a small number of early-onset cases that are afflicted because of high penetrant single gene mutations in *APP*, *PSEN1*, and *PSEN2* genes, AD is genetically heterogeneous, especially the late-onset form having a polygenic or oligogenic risk inheritance. Since the identification of *APOE* as the most significant risk factor for late-onset AD in 1993, the path to the discovery of additional AD risk genes had been arduous until 2009 when the use of large genome-wide association studies opened up the discovery gateways that led the identification of ~ 95 additional risk loci from 2009 to early 2022. This article reviews the history of AD genetics followed by the potential molecular pathways and recent application of functional genomics methods to identify the causal AD gene(s) among the many genes that reside within a single locus. The ultimate goal of integrating genomics and functional genomics is to discover novel pathways underlying the AD pathobiology in order to identify drug targets for the therapeutic treatment of this heterogeneous disorder.

**Supplementary Information:**

The online version contains supplementary material available at 10.1007/s13311-021-01152-0.

## Introduction

Alzheimer’s disease (AD) is a complex and multifactorial neurodegenerative disease and the leading cause of dementia among elderly, accounting for 60–80% of all dementia cases. However, a small number of people (5–10%) develop AD at a younger age, and due to this variation in age-at-onset (AAO), the disease is classified into early-onset (EOAD) with AAO < 60 years and late-onset (LOAD) with AAO ≥ 60 years. Deposition of amyloid-beta (Aβ) plaques and the formation of neurofibrillary tangles (tau pathology) in the brain are required for the neuropathological diagnosis of AD [1]. Approximately half of the cases with AD dementia have solely AD brain pathology, and the remaining cases have coexisting pathological brain changes of AD and other dementia(s), like vascular dementia and Lewy body dementia. Such subjects with mixed pathologies are termed having mixed dementia [2]. Due to its long clinical course, transitioning from mild to moderate and severe stage, AD is a major public health problem in the USA and worldwide. On average, a person with AD can survive 4–8 years after the diagnosis, but some can survive as long as 20 years. There are over 50 million people worldwide living with AD or dementia, and this number is projected to increase 152 million in 2050 [3]. Currently, there are 6.2 million AD cases in the USA [2]. The estimated annual cost for caring of AD patients in the USA is projected to be $1.1 trillion in 2050 when the number of AD cases would reach to ~ 14 million, if no medical breakthroughs are found.

Among the multiple known risk factors for AD, the strongest evidence is for age, biological (gender), and genetic differences. Although AD is not a normal part of aging, older age is the greatest risk factor for AD. The prevalence of AD increases dramatically after age 60: 5% in age group 65–74, 14% in age group 75–85, and 35% in age group 85 and older [2]. Currently, about 58 million Americans are age 65 and older and the Bureau of the Census estimates that this number will be 58 million by the year 2050. Due to this alarming increase in the elderly population and with the possibility that a large fraction of this elderly population would suffer from AD, it is essential to understand the causes of AD so that effective preventative measures could be devised. In fact, the oldest members of the baby-boom generation of Americans (born between 1946 and 1964) have already turned age 75 in 2021. The prevalence of AD is higher in women than men; almost two-thirds of the AD cases are women [2]. The estimated lifetime risk for AD among Americans at age 45 or 65 is 20% in women and 10% in men [4]. In addition to the biological difference in men and women, this gender difference could be due to “survival effect” (women live longer than men and the older age is the greatest risk for AD) or “survival bias” (men who survive beyond age 60 have healthier cardiovascular profile than women have and thus have lower risk of AD) [2].

The role of genetics in the etiology is well recognized in both EOAD and LOAD. Mutations in three casual genes for EOAD have been identified (*APP*, *PSEN1*, and *PSEN2*) that follow the autosomal dominant inheritance pattern. LOAD that comprises 90–95% of the cases is genetically more complex with heritability estimates from 58 to 70% [5, 6]. *APOE* was identified as the first susceptibility gene for LOAD in 1993, which was followed by a plethora of linkage, and positional and biological candidate gene approaches to identify additional genes but without a success [7, 8]. Until 2009, *APOE* was the only established susceptibility gene for LOAD. Since 2009, substantial progress has been made in LOAD genetics via large-scale case–control genome-wide association studies (GWAS) as well as meta-analyses of GWAS that have led to the identification numerous susceptibility loci. Recent applications of whole-exome microarray, whole-exome sequencing (WES), and whole-genome sequencing (WGS) have also identified rare variants in additional novel LOAD genes. However, the common single nucleotide polymorphism (SNP)-based heritability from known AD genes ranges from 13 to 33% [9–11], indicating that much of the AD genetic variance remains unexplained. One of the likely explanations of relatively low genetic variance observed with known LOAD loci in case–control studies is the inclusion of “non-AD dementia” subjects in the case group based on clinical AD diagnosis that is prone to high misclassification, and the control group may include many “presumptive AD cases” because the preclinical stage of AD begins decades before the initial clinical symptoms of AD [12, 13]. In order to overcome this misclassification and to better understand the underlying mechanisms of AD clinical expression, the 2018 research framework proposed by the National Institute on Aging and Alzheimer’s Association defines AD as a biological construct that is identified by biomarkers in living people [14].

The purpose of this review is to provide an update on the genomics landscape of AD and then discuss the ongoing efforts in integrating GWAS findings in LOAD with functional genomics in order to identify putative functional genes and molecular pathways.

## Early-Onset Alzheimer’s Disease

Approximately 5–10% of all AD cases are classified as EOAD, of which 35–65% are familial (FEOAD) and the remaining are sporadic [15, 16]. Of the FEOAD cases, about 10–15% follow an autosomal dominant inheritance pattern [17] due to mutations in three known genes: *APP*, *PSEN1*, and *PSEN2*. Approximately 90% of the remaining FEOAD cases are most likely due to autosomal recessive inheritance pattern [6]. Mutations in the three-causal EOAD genes regulate the production, aggregation, and degradation of beta-amyloid peptides (Aβs), which are primary components of amyloid plaques.

**Fig. 1:**
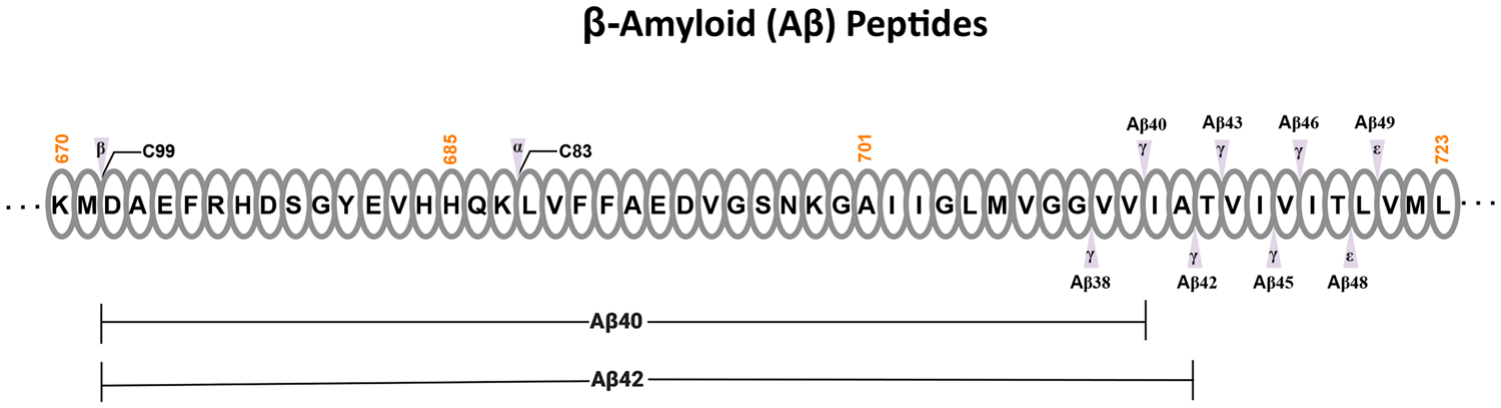
Generation of β-amyloid (Aβ) peptides. The APP amino acid sequence from position 670 to 723 is shown, including the transmembrane region from amino acid 701 to 723. The cleavage sites of α-, β-, and γ-secretases are labelled. Following the generation of APP-C99 by β-secretase, it is cleaved by γ-secretase through its endopeptidase activity (ε-site) to generate Aβ48 or Aβ49 peptide, which is then further cleaved sequentially by the C-terminal peptidase activity of γ-secretase to produce Aβ45, Aβ42, and Aβ38 peptides from Aβ48 (shown at the right bottom end) or Aβ46, Aβ43, and Aβ40 peptides from Aβ49 (shown at the right top end). The full lengths of Aβ40 and Aβ42 peptides are illustrated at the bottom. The start position of APP-C83 following the cleavage with α-secretase is also shown

Aβs are derived from the amyloid precursor protein (APP), which is a transmembrane protein consisting of an amino-terminal domain of 699 amino acids (in the longest APP-770) in the extracellular or intraluminal region, a short transmembrane region of 24 amino acids (from 700 to 723), and a 47-amino acid long intracellular domain known as AICD (APP intracellular domain). APP undergoes three alternative proteolytic cleavages by α-, β-, and γ-secretases within or surrounding the Aβ sequence to generate either smaller fragments in the non-amyloidogenic pathway or longer fragments in the amyloidogenic pathways [18]. APP is first cleaved by either α-secretase at amino acid 687 or β-secretase (BACE1) at amino acid 671 to generate a transmembrane carboxyl fragment of APP-C83 or APP-C99, respectively (Fig. 1). APP-C83 or APP-C99 is then cleaved by the transmembrane aspartyl protease γ-secretase to generate the smaller harmless peptides or the longer pathogenic peptides, respectively [19]. Initially, APP-C99 is cleaved by γ-secretase through its endopeptidase activity (ε-site) to generate Aβ48 or Aβ49 peptide, which is then further cleaved sequentially by the carboxypeptidase activity of γ-secretase to produce Aβ45, Aβ42, and Aβ38 peptides from Aβ48 or Aβ46, Aβ43, and Aβ40 peptides from Aβ49 (Fig. 1). Of these peptides, Aβ40 is the most common and Aβ42, which constitutes approximately 10% of the total Aß, is particularly associated with the development of AD [20]. With the extra two hydrophobic amino acids, Aβ42 is more prone to aggregation than Aβ40 and is found abundantly in neuritic plaques and amyloid-bearing microvessels.

Human γ-secretase complex consists of four protein subunits: presenilin (PSEN), presenilin enhancer 2 (PEN2), anterior pharynx-defective-1 (APH1), and nicastrin (NCT). The catalytic activity of γ-secretase is determined by PSEN, which exists in two isoforms (PSEN1 and PSEN2). APH1 stabilizes the complex and has two isoforms (APH1A and APH1B), PEN2 is essential for γ-secretase maturation, and NCT plays a role in substrate binding [19–22].

A large number of high-penetrant mutations have been identified in the *APP*, *PSEN1*, and *PSEN2* genes that regulate Aβs production (especially Aβ40 and Aβ42) and cause EOAD. As of October 2021, Alzforum lists 50, 309, and 55 AD-associated mutations in *APP*, *PSEN1*, and *PSEN2*, respectively (www.alzforum.org/mutations). Altogether, pathogenic mutations in these three genes account for approximately 7% of the EOAD cases, with a 6% contribution from *PSEN1* and ~ 1% from the other two genes [23]. The majority of the *APP* mutations are missense that occurs in or around the Aβ sequence and lead to either overproduction of total Aβ or increased Aβ42/Aβ40 ratio. With the exception of two mutations (p.Ala673Val and one amino acid deletion, p.Glu693Δ), which are autosomal recessive, all other *APP* mutations are autosomal dominant [16, 24]. Two de novo* APP* duplications have also been described [23]. Overproduction of Aβ is also a key feature in Down syndrome patients who have three copies of the *APP* containing chromosome 21 and develop a neuropathology that is indistinguishable from AD [24]. These findings tend to support the amyloid cascade hypothesis that deposition of Aβ in the brain is the initiating factor in the pathogenesis of AD [25].

The majority of the *PSEN1* and *PSEN2* mutations are missense with autosomal dominant inheritance, although some autosomal recessive and de novo mutations have also been described [23]. Mutations in the *PSEN1* and *PSEN2* genes increase Aβ42, but decrease Aβ40, resulting in an increased Aβ42/Aβ40 ratio. These mutations also shift Aβ40 and Aβ42 to longer and more harmful species. Increased Aβ42/Aβ40 ratio has also shown to be associated with disease progression, as reflected in early AAO in familial AD. However, a comprehensive in vitro analysis of the effect of 138 *PSEN1* pathogenic mutations on γ-secretase activity in the production of Aβ42 and Aβ40 found no significant correlation between the Aβ42/Aβ40 ratio and the mean AAO [26]. Furthermore, about 90% of the examined 138 *PSEN1* mutations resulted in reduced production of Aβ42 and Aβ40 and 10% of them lead to decreased Aβ42/Aβ40 ratio in the experimental system used in the study. These findings appear to contradict the amyloid hypothesis and suggest the possible involvement of multiple mechanisms in the etiology of AD where the amyloid pathway may just constitute one of them [26]. This also highlights the need to assess the functional nature of all the *APP*, *PSEN1*, and *PSEN2* mutations because the pathogenicity of most of these mutations is yet to be examined.

To date, no mutations have been described to be associated with EOAD either in the *ADAM10* or *ADAM17* (key components of α-secretase complex) and *BACE1* genes or the genes coding for the other members of the γ-secretase complex (*PEN2*, *APH-1*, *NCT*). However, rare missense mutations in the *SORL1* gene have been suggested to play an important role in subset of FEOAD [27, 28].

### Role of Early-Onset AD Genes in Late-Onset AD

Despite the high penetrance of *APP*, *PSEN1*, and *PSEN2* mutations in EOAD, their role in LOAD susceptibility was not clear, and so was the case with other genes involved in the regulation of APP metabolism and/or Aβ generation. This was changed, however, in 2009 when two rare missense mutations in *ADAM10* (p.Gln170His; p.Arg181Gly) were reported to cosegregate in LOAD families [29]. These mutations attenuated α-secretase activity of ADAM10 and increased Aβ plaques and reactive gliosis in transgenic mice [30]. In 2012, two studies reported rare risk and protective variants of *APP* in LOAD. In the first study, a rare pathogenic *APP* coding variant (p.Asn660Tyr) exhibiting high disease penetrance was reported in multiple members of an LOAD family, which was absent in 1346 controls and 12,481 subjects not enriched for AD [31]. In the second study, a rare but widely distributed *APP* coding variant (p.Ala673Thr/rs63750847) was found to be protective against sporadic LOAD and cognitive impairment in non-AD elderly individuals among Icelanders, Finnish, Norwegian, and Swedish populations [32]. The minor allele frequency (MAF) of this variant was 0.13% in AD cases compared with 0.45% in population controls and 0.62% in population controls aged 85 or greater. Subsequent large LOAD studies in US Whites found either no or only sporadic examples of p.Ala673Thr [33, 34], indicating its confinement to individuals of the origin from the Nordic countries. This variant is also absent among Chinese [35]. The p.Ala673Thr variant is present at the same site as the autosomal recessive p.Ala673Val pathogenic mutation and is adjacent to the BACE1 cleavage site at position 671. The protective p.Ala673Thr mutation is associated with reduced production of amyloidogenic Aβ peptides by about 40%, and the generated Aβ is less prone to aggregation [32, 35]. This protective variant was also detected in a 105-year-old Finnish demented subject who showed little Aβ pathology [36].

In addition to rare variants, recent large GWAS have identified common variants in the *ADAM10*, *ADAM17*, *APH1B*, and *APP-ADAMTS1* gene regions to be associated with LOAD susceptibility (discussed later), further providing support to the amyloid cascade hypothesis in at least subset of the LOAD patients.

## Late-Onset Alzheimer’s Disease

### *APOE* Polymorphism

**Fig. 2:**
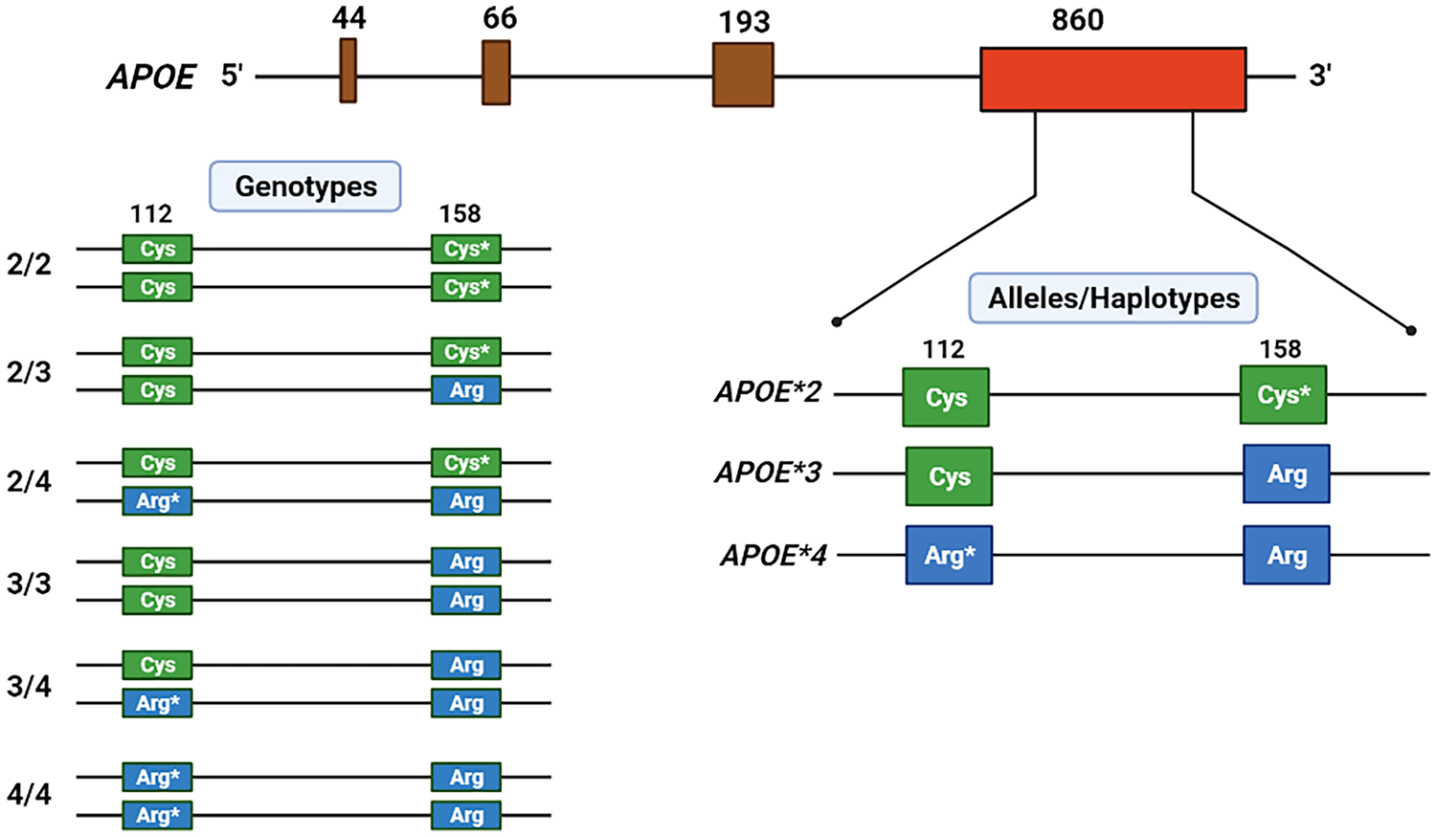
Structure of the *APOE* gene with four exons (top). Non-synonymous mutations at codon 112 (Cys112Arg) and codon 158 (Arg158Cys) in exon 4 code for three common alleles/haplotypes: APOE*2, APOE*3, and APOE*4 (bottom right), resulting in six genotypes (bottom left). The most common APOE*3 allele has Cys at position 112 and Arg at position 158 in the ApoE protein. The amino acid change in APOE*4 at codon 112 is indicated by Arg*, and the amino acid change in APOE*2 at codon 158 is indicated by Cys*

The strongest risk factor for LOAD is the common three-allele *APOE* polymorphism: APOE*2, APOE*3, and APOE*4 [37, 38], resulting in six genotypes (2/2,2/3,2/4,3/3,3/4,4/4). This polymorphism is characterized by missense mutations at the first base of codon 112 and codon158 (Fig. 2). APOE*3 is the most common allele and code for Cys (TGC) at position 112 and Arg (CGC) at position 158. APOE*4 and APOE*2 differ from APOE*3 by having Arg (**C**GC) at position 112 and Cys (**T**GC) at position 158, respectively. Due to strong linkage disequilibrium (LD) between the two sites, the three alleles also define three rather than the four expected haplotypes: E3 has Cys at position 112 and Arg at position 158, E4 has Arg at both positions, and E2 has Cys at both positions. The expected fourth haplotype having Arg at position 112 (E*4 allele) and Cys at position 158 (E*2 allele) has been observed only rarely [39]. These structural differences in *APOE* alleles have profound effect on the function of the ApoE protein in lipid metabolism/cardiovascular function and in determining AD risk [37, 40–45]. Although APOE*4 is associated with adverse plasma lipid profile (high LDL-cholesterol, high ApoB, low plasma ApoE levels) and high risk of heart disease and AD, the effect of APOE*2 on these traits is in opposite direction. APOE*4 is also associated with AD-related proteinopathies: Aβ, tau, α-synuclein, and TDP-43 [42, 43]. The differential *APOE* allelic effect on AD risk is likely regulated in large part due to its impact on AD-related proteinopathies. APOE*4 may also have direct pathologic effects on neurons and the blood–brain barrier function independent of its effects on amyloid and tau pathologies [43].

The frequencies of three *APOE* alleles and the proportion of people who carry them (genotype carriers) differ significantly among the major human racial groups (Table 1). The aboriginal populations of Australia and America seem to have no or only sporadic presence of the APOE*2 allele, which is present at an allele frequency of about 4% in Blacks and 8% in most other populations. Likewise, the average occurrence of APOE*4 allele frequency (genotype carriers) is 7% (14%) in Asians–Japanese/Chinese; 14% (25%) in Whites; 26–37% (40–50%) in Blacks, Australian Aborigines, and Pacific populations; and 23% (40%) in Eskimos. Although the prevalence of AD is not well documented in the aboriginal populations of Australia and America as well as in the Pacific populations, these populations seem to be at high risk of developing AD by having a very high frequency of APOE*4 carriers should their life expectancy increases, as seen among the European or European-derived White populations.

**Table 1:** *APOE* allele frequencies in major racial groups*

Population	APOE*2	APOE*3	APOE*4
**Whites**			
US Whites	0.07	0.79	0.14
Germans	0.08	0.77	0.15
French	0.13	0.74	0.13
Finns	0.04	0.73	0.23
Dutch	0.08	0.75	0.17
**Africans**			
American blacks	0.03	0.71	0.26
Nigerians	0.03	0.67	0.30
Sudanese	0.08	0.63	0.29
**Asians**			
Japanese	0.08	0.85	0.07
Chinese	0.10	0.83	0.07
**Oceanians**			
New Guineans	0.14	0.49	0.37
Australian Aborigines	0.00	0.74	0.26
Polynesians	0.11	0.63	0.26
**Native Americans**			
Amerindians	0.00	0.82	0.18
Mayans	0.00	0.91	0.09
Eskimos	0.01	0.76	0.23

^*^Adapted from Kamboh 1995 [37]

APOE*4 is a significant risk factor for AD and shows gene dosage effect on AD risk in populations with diverse racial/ethnic backgrounds. However, APOE*4 is neither necessary nor sufficient to develop AD, indicating the involvement of additional genetic factors that can modify the risk of AD. There is also considerable inter-racial variation in the gene dosage effect of APOE*4 on AD risk, as measured in odds ratio (OR). The ORs with one and two copies APOE*4 in major racial groups are as follows: 3.5 and 14.5 in Whites; 1.1–2.2 and 2.2–5.7 in African Americans; 3.1 and 11.8 in Chinese; and 5.6 and 33.1 in Japanese [38, 46–48]. The relatively higher ORs in one Japanese study need to be confirmed in larger Japanese samples because this study used only 336 AD cases and thus may not be representative of the Japanese population. However, the findings of low ORs in African Americans are intriguing despite that the frequency of APOE*4 is almost twice in Africans compared to Whites. This difference may be due to variation in environmental and cultural factors, or it may represent additional genetic variation present in the *APOE* region, which is protective in Africans. Indeed, it has been suggested that the genomic region surrounding *APOE* with African background reduces the risk for APOE*4 carriers [48]. In addition to the common *APOE* polymorphism, which explains about 25% of the genetic variance of AD [9], rare coding and noncoding variants in *APOE* have also been implicated with the risk of AD [49–51].

### Genome-Wide Association Studies

**Fig. 3:**
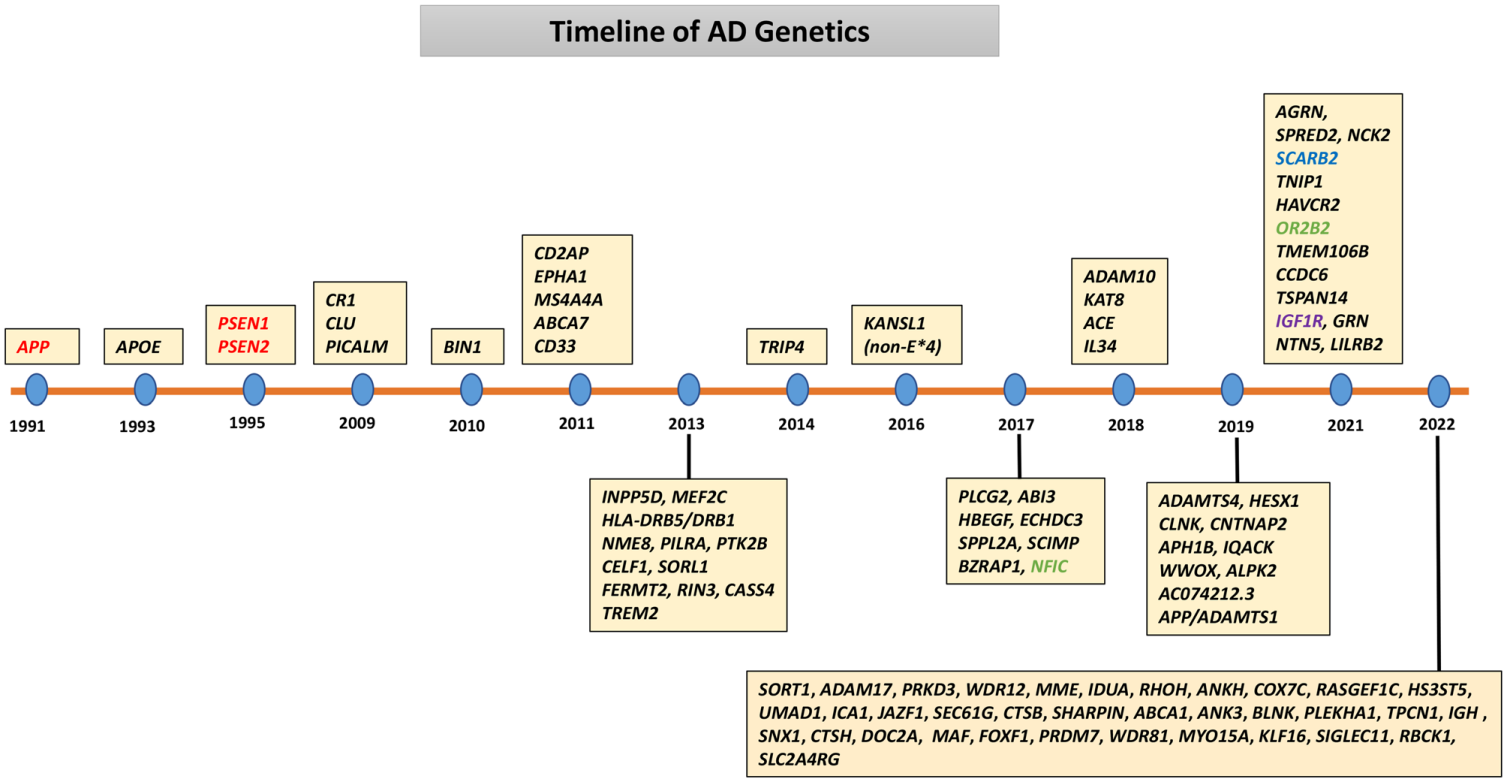
Timeline of the discovery of AD genes/loci. Red color labelled *APP*, *PSEN1*, and *PSEN2* genes are for early-onset AD. Green color labelled *NFIC* and *OR2B2* loci were discovered in transethnic GWAS. Purple color labelled *IGF1R* locus is unique to African Americans and blue color labelled *SCARB2* locus is unique to Japanese. The remaining loci were discovered in Europeans/Whites

In order to identify additional genes/loci for LOAD, genome-wide association studies (GWAS) were initiated soon after the availability of chip microarrays because this approach is hypothesis free and conceptually would identify all known and unknown genes. However, with the exception of the *APOE* region, no other genome-wide significant (GWS) associations (*p* < 5E − 08) were identified in earlier GWAS published during 2007–2008 [52–59]. The GWS threshold of *p* < 5E − 08 (0.05/1,000,000) is widely applied to avoid false-positive findings in GWAS comprising approximately 1 million independent association tests. These early negative findings were due to the use of modest sample size and suggested that the effect sizes of yet to be discovered variants were very small as compared with the large effect of APOE*4 and that it would require the use of large number of cases and controls in order to detect small effect sizes. Indeed, the recent use of large GWAS and meta-analyses of GWAS has led to the identification of ~ 95 loci for LOAD during the period of 2009 to early 2022. The timeline of AD genetics is illustrated in Fig. 3, and the history and outcomes of GWAS are summarized in the below sections and in Table 2. It is worth noting that the gene names given in Fig. 3 and Table 2 represent the closest gene to the lead SNP, not necessarily the causal gene in the locus, and that the gene name used to refer to a locus can change between GWAS depending on the lead variant and its corresponding closest gene. Like other diseases, the focus of the vast majority of genomic studies in LOAD has been on European or European-derived White populations.

**Table 2:** Genome-wide significant AD-associated loci

**Chr**	**Position (bp)**^**a**^	**Lead SNP**	**Nearest gene**	**Major/minor allele**	**MAF**	**OR (95% CI)**^**b**^	***p*-value**
1	1,049,997	rs113020870	*AGRN*	C/T	0.004	1.07 (1.05–1.10)	3.8E − 08
1	161,185,602	rs4575098 (3′UTR)	*ADAMTS4*	G/A	0.239	1.02 (1.01–1.02)	2.1E − 10
1	109,345,810	rs141749679 (p.Lys165Glu)	*SORT1*	T/C	0.004	1.38 (1.24–1.54)	7.5E − 09
1	207,518,704	rs6656401	*CR1*/*CD55*	G/A	0.197	1.18 (1.14–1.22)	5.7E − 24
2	9,558,882	rs72777026	*ADAM17*	A/G	0.144	1.06 (1.04–1.08)	2.7E − 08
2	37,304,796	rs17020490	*PRKD3*	T/C	0.145	1.06 (1.04–1.08)	3.3E − 09
2	65,381,229	rs268134	*SPRED2*	A/G	0.250	0.94 (0.92–0.96)	1.5E − 08
2	105,749,599	rs143080277	*NCK2*	T/C	0.004	1.45 (1.34–1.63)	1.3E − 12
2	127,135,234	rs6733839	*BIN1*	C/T	0.407	1.20 (1.17–1.23)	2.1E − 44
2	202,878,716	rs139643391 (3′UTR)	*WDR12*	TC/T	0.131	0.94 (0.92–0.96)	1.1E − 08
2	233,117,202	rs10933431	*INPP5D*	C/G	0.223	0.91 (0.88–0.94)	3.4E − 09
3	57,192,122	rs184384746	*HESX1*	C/T	0.002	1.21 (1.14–1.30)	1.2E − 08
3	155,069,722	rs16824536	*MME*	G/A	0.054	0.92 (0.89–0.95)	3.6E − 08
	155,084,189	rs61762319 (Met8Val)	*MME*	A/G	0.026	1.16 (1.11–1.21)	2.2E − 11
4	993,555	rs3822030	*IDUA*	T/G	0.429	0.95 (0.94–0.96)	8.3E − 12
4	11,025,995	rs4351014	*CLNK*	T/C	0.265	0.93 (0.91–0.95)	2.6E − 11
4	40,197,226	rs2245466 (5′UTR)	*RHOH*	C/G	0.343	1.05 (1.03–1.06)	1.2E − 09
4	76,217,307	rs920608	*SCARB2/FAM47E*	A/C	0.038	0.65 (0.57–0.75)	5.3E − 09^c^
5	14,724,304	rs112403360	*ANKH*	T/A	0.073	1.09 (1.06–1.12)	2.3E − 09
5	86,927,378	rs62374257	*COX7C*	T/C	0.23	1.07 (1.05–1.09)	1.4E − 15
5	88,927,603	rs190982	*MEF2C*	A/G	0·408	0.92 (0.89–0.95)	3.2E − 08
5	140,335,105	rs2074612	*APBB3*/*HBEGF*	C/T	0.438	1.08 (1.05–1.11)	8.0E − 09
5	151,052,827	rs871269	*TNIP1*	C/T	0.32	0.98 (0.97–0.99)	1.4E − 09
5	157,099,320	rs6891966	*HAVCR2*	G/A	0.23	0.98 (0.97–0.99)	7.9E − 10
5	180,201,150	rs113706587	*RASGEF1C*	G/A	0.11	1.09 (1.07–1.12)	2.2E − 16
6	27,915,491	rs1497525	*OR2B2*	C/A	0.05–0.08	1.34 (1.15–1.56)	2.1E − 08^d^
6	32,592,048	rs34855541	*HLA-DRB1*/*DRB5*	A/G	0.135	0.90 (0.87–0.92)	9.5E − 15
6	41,161,514	rs75932628 (p.Arg47His)	*TREM2*	C/T	0.008	2.01 (1.65–2.44)	2.7E − 15
6	47,520,026	rs10948363	*CD2AP*	A/G	0.266	1.10 (1.07–1.13)	5.2E − 11
6	114,291,731	rs785129	*HS3ST5*	C/T	0.35	1.04 (1.03–1.06)	2.4E − 09
7	7,817,263	rs6943429	*UMAD1*	C/T	0.42	1.05 (1.03–1.06)	1.0E − 10
7	8,204,382	rs10952097	*ICA1*	C/T	0.114	1.07 (1.05–1.10)	6.8E − 09
7	12,229,132	rs5011436	*TMEM106B*	A/C	0.41	1.02 (1.01–1.02)	2.7E − 09
7	28,129,127–28,129,134	rs1160871	*JAZF1*	GTCTT/G	0.222	0.95 (0.93–0.97)	9.8E − 09
7	37,801,932	rs2718058	*GPR141*/*NME8*	A/G	0·373	0.93 (0.90–0.95)	4.8E − 09
7	54,873,635	rs76928645	*SEC61G*	C/T	0.103	0.93 (0.91–0.95)	1.6E − 10
7	100,374,211	rs1859788 (p.Gly78Arg)	*PILRA*	G/A	0.321	0.98 (0.98–0.99)	3.3E − 18
7	143,402,040	rs10808026	*EPHA1*/*ZYX*	C/A	0.199	0.90 (0.88–0.93)	1.3E − 10
7	146,252,937	rs114360492	*CNTNAP2*	C/T	< 0.001	1.19 (1.12–1.26)	2.1E − 09
8	11,844,613	rs1065712 (3′UTR)	*CTSB*	G/C	0.053	1.09 (1.06–1.12)	1.9E − 09
8	27,362,470	rs73223431	*PTK2B*	C/T	0.367	1.10 (1.07–1.13)	6.3E − 14
8	27,610,169	rs9331896	*CLU*	T/C	0.387	0.88 (0.85–0.90)	4.6E − 24
8	144,103,704	rs34173062	*SHARPIN*	G/A	0.081	1.13 (1.09–1.16)	1.7E − 18
9	104,903,697	rs1800978	*ABCA1*	C/G	0.13	1.06 (1.04–1.08)	1.6E − 09
10	11,678,309	rs7920721	*ECHDC3*	A/G	0.389	1.08 (1.05–1.11)	2.3E − 09
10	60,025,170	rs7068231	*ANK3*	G/T	0.403	0.95 (0.94–0.96)	3.3E − 13
10	59,886,075	rs1171814	*CCDC6*	G/T	0.481	0.95 (0.93–0.97)	3.8E − 08
10	80,520,381	rs1878036	*TSPAN14*	T/G	0.207	1.07 (1.05–1.10)	2.7E − 09
10	96,266,650	rs6584063	*BLNK*	A/G	0.043	0.89 (0.86–0.92)	6.7E − 11
10	122,413,396	rs7908662	*PLEKHA1*	A/G	0.467	0.96 (0.95–0.97)	2.6E − 09
11	47,358,789	rs3740688	*SPI1*/*CELF1*	T/G	0.448	0.92 (0.89–0.94)	5.4E − 13
11	60,169,453	rs7933202	*MS4A2*	A/C	0.391	0.89 (0.87–0.92)	1.9E − 19
11	86,156,833	rs10792832	*PICALM*	G/A	0.358	0.87 (0.85–0.89)	9.3E − 26
11	121,564,878	rs11218343	*SORL1*	T/C	0.040	0.80 (0.75–0.85)	2.9E − 12
12	113,281,983	rs6489896	*TPCN1*	T/C	0.076	1.08 (1.05–1.10)	1.8E − 09
14	52,924,962	rs17125924	*FERMT2*	A/G	0.099	1.12 (1.08–1.15)	1.3E − 11
14	92,460,608	rs10498633	*RIN3*/*SLC24A4*	G/T	0.217	0.91 (0.88–0.94)	5.5E − 09
14	105,761,758	rs7157106	*IGH* cluster	G/A	0.36	1.05 (1.03–1.07)	2.0E − 08
	106,665,591	rs10131280	*IGH* cluster	G/A	0.133	0.94 (0.92–0.96)	4.3E − 10
15	50,709,337	rs59685680	*SPPL2A*	T/G	0.198	0.92 (0.89–0.95)	7.3E − 09
15	58,753,575	rs593742	*ADAM10*	A/G	0.298	0.93 (0.91–0.95)	2.8E − 11
15	63,277,703	rs117618017 (p.Thr27Ile)	*APH1B*	C/T	0.139	1.09 (1.06–1.12)	1.5E − 08
15	64,131,307	rs3848143	*SNX1*	A/G	0.22	1.05 (1.04–1.07)	8.4E − 11
15	64,433,291	rs74615166	*TRIP4*	T/C	0.02	1.31 (1.19–1.44)	9.7E − 09
15	78,936,857	rs12592898	*CTSH*	G/A	0.133	0.94 (0.92–0.96)	4.2E − 09
15	97,449,455	rs570487962	*IGF1R*	Rare variants	0.01	0.10 (0.05–0.22)	1.6E − 09^e^
16	19,796,841	rs7185636	*IQCK*	T/C	0.156	0.92 (0.89–0.95)	2.4E − 08
16	30,010,081	rs1140239	*DOC2A*	C/T	0.379	0.94 (0.93–0.96)	2.6E − 13
16	31,111,250	rs889555	*KAT8*	C/T	0.29	0.95 (0.94–0.97)	3.2E − 08
16	70,660,097	rs4985556 (p.Tyr213Ter)	*IL34*	C/A	0.088	1.09 (1.05–1.12)	3.7E − 08
16	79,321,960	rs62039712	*WWOX*	G/A	0.094	1.16 (1.09–1.24)	3.7E − 08
16	79,574,511	rs450674	*MAF*	T/C	0.373	0.96 (0.95–0.98)	3.2E − 08
16	81,908,423	rs72824905 (p.Pro522Arg)	*PLCG2*	C/G	0.009	0.68 (0.60–0.77)	5.4E − 10
16	86,420,604	rs16941239	*FOXF1*	T/A	0.029	1.13 (1.08–1.17)	1.3E − 08
16	90,103,687	rs56407236	*PRDM7*	G/A	0.069	1.11 (1.08–1.14)	6.5E − 15
17	1,728,047	rs35048651 (5′UTR)	*WDR81*	TGAG/T	0.214	1.06 (1.04–1.08)	7.7E − 11
17	5,233,752	rs7225151	*RABEP1*/*SCIMP*	G/A	0.118	1.10 (1.07–1.13)	6.1E − 12
17	18,156,140	rs2242595	*MYO15A*	G/A	0.112	0.94 (0.92–0.96)	1.1E − 09
17	44,364,976	rs708382	*GRN*	T/C	0.39	1.02 (1.01–1.02)	2.0E − 09
17	46,275,856	rs2732703	*KANSL1*/*LRRC37A*	T/G	0.13	0.73 (0.65–0.81)	5.8E − 09^f^
17	49,219,935	rs616338 (p.Ser209Phe)	*ABI3*	C/T	0.008	1.43 (1.28–1.60)	4.6E − 10
17	58,320,645	rs2526380	*BZRAP1*/	G/C	0.44	0.97 (0.96–0.98)	2.6E − 08
	58,331,728	rs2632516	*TSPOAP1-ASI*	G/C	0.44–0.60	0.92 (0.91–0.94)	4.4E − 08^g^
17	63,460,787	rs138190086	*ACE/CYB561*	G/A	0.017	1.25 (1.16–1.35)	1.9E − 09
18	58,522,227	rs76726049	*ALPK2*	T/C	0.011	1.06 (1.04–1.08)	3.3E − 08
19	1,056,493	rs3752246	*ABCA7*	C/G	0.182	1.15 (1.11–1.18)	3.1E − 16
19	1,854,255	rs149080927 (3′UTR)	*KLF16*	GC/G	0.48	1.05 (1.04–1.07)	5.1E − 10
19	3,405,594	rs9749589	*NFIC*	T/A	0.02–0.16	0.76 (0.69–0.83)	1.5E − 08^h^
19	44,908,684	rs429358 (p.Cys112Arg)	*APOE*	T/C	0.216	3.32 (3.20–3.45)	1.2E − 881
19	45,738,583	rs76320948	*AC074212.3*	C/T	0.046	0.97 (0.96–0.98)	4.6E − 08
19	48,710,247	rs2452170	*NTN5*	A/G	0.47	0.99 (0.99–1.00)	1.7E − 08
19	49,950,060	rs9304690	*SIGLEC11*	C/T	0.24	1.05 (1.03–1.07)	4.7E − 09
19	51,224,706	rs3865444 (5′UTR)	*CD33*	C/A	0.336	0.99 (0.98–0.99)	6.3E − 09
19	54,313,903	rs1761461	*LILRB2*	A/C	0.49	1.01 (1.01–1.02)	1.6E − 09
20	413,334	rs1358782	*RBCK1*	G/A	0.246	0.95 (0.94–0.97)	1.6E − 08
20	56,423,488	rs6014724	*CASS4*	A/G	0.088	0.89 (0.87–0.93)	1.1E − 10
20	63,743,088	rs6742	*SLC2A4RG*	C/T	0.221	0.95 (0.93–0.97)	2.6E − 09
21	26,784,537	rs2830500	*APP*/*ADAMTS1*	C/A	0.336	0.93 (0.91–0.95)	2.6E − 08

Chr = chromosome, MAF = minor allele frequency and the effect allele

^a^GRCh38 assembly

^b^Odds ratio with 95% confidence interval

^c^Among Japanese

^d^Transethnic GWAS including Japanese and Europeans

^e^Among African Americans

^f^Among non-APOE*4

^g^Transethnic GWAS in which MAF varied from 0.44 to 0.60 in different ethnic groups

^h^Transethnic GWAS in which MAF varied from 0.02 to 0.16 in different ethnic groups, *p*-value represents interaction term (*NFIC*/rs9749589 × APOE*4)

### GWAS in Europeans/Whites

In 2009, two large GWAS published back-to-back in *Nature Genetics* identified three novel GWS loci (*CLU*, *PICALM*, and *CR1*) in addition to *APOE* [60, 61]. The combined sample in the first study, which was derived from a collaborative consortium Genetic and Environmental Risk in Alzheimer’s Disease (GERAD) from Europe and the USA, was 16,152 subjects (5964 cases, 10,188 controls) from the discovery and replication stages and identified the *CLU* (also known as *APOJ*) and *PICALM* loci [60]. The second study also identified *CLU* as well as *CR1* in a total discovery and replication sample of 14,636 subjects (6010 cases, 8625 controls) from the European Alzheimer’s Disease Initiative (EADI) [61]. Another novel locus, *BIN1*, was identified in 2010 in a three-stage analysis of new and previously published GWAS on 35,336 subjects (8371 cases, 26,965 controls) derived from the Cohorts for Heart and Aging Research in Genomic Epidemiology (CHARGE) consortium, Translational Genomics Research Institute (TGEN), Mayo AD GWAS, EADI, and GERAD consortium [62]. In 2011, five new loci (*CD2AP*, *EPHA1*, *MS4A4A/MS4A6A*, *ABCA7*, and *CD33*) were reported in two back-to-back publications in *Nature Genetics* [63, 64]. The first study comprising 59,716 individuals (19,870 cases, 39,846 controls) from GERAD, GERAD 2, EADI, EADI 2, Mayo 2, CHARGE, Alzheimer’s Disease Neuroimaging Initiative (ADNI), and TGEN (collectively called GERAD +) in a 3-stage study design identified the *ABCA7* and *MS4A6A/MS4A4E* loci. The second study used a 2-stage study consisting of 22,771 subjects (11,840 cases, 10,931 controls) derived from 12 cohorts and the U.S. National Institute on Aging (NIA)–funded Alzheimer Disease Centers, as part of the Alzheimer Disease Genetics Consortium (ADGC), and identified the *MS4A4/MS4A6E* and *EPHA1* loci. Furthermore, two loci (*CD33* and *CD2AP*) were identified by combining GERAD + and ADGC results: a) GERAD + and 19,072 ADGC subjects (9980 cases, 9090 controls), and b) ADGC and 31,658 GERAD + samples (6992 cases, 24,666 controls). The different lead SNPs noted at the *MS4A* (membrane-spanning 4-domains subfamily A) locus on chromosome 11 in these two studies detected the same signal due to the strong LD in this region that contains six of the 16 *MS4A* genes. Noteworthy, evidence for suggestive association with *CD33* and *EPHA1* was also reported earlier [56, 62], but they achieved GWS only using much larger sample size. The previously reported GWS *EXOC3L2* locus on chromosome 19 [62], which is within 300 kb of *APOE*, was not found to be significant after adjusting for the effect of *APOE* [64]. The number of total loci, including *APOE*, for LOAD reached to 10 in 2011.

Realizing that even much larger sample size is needed to identify additional loci, investigators from the ADGC, CHARGE, EADI, and GERAD consortia along with additional investigators from Europe and the USA joined hands and established the International Genomics of Alzheimer’s Project (IGAP) comprising a total sample of 74,046 individuals (25,580 cases and 48,466 controls) [65]. The IGAP conducted a meta-GWAS analysis on 54,162 samples in stage 1 and genotyped top SNPs in additional 19,884 samples in stage 2 and reported the identification of 11 new susceptibility loci in 2013: *INPP5D*, *MEF2C*, *HLA-DRB5/DRB1*, *NME8*/*GPR141*, *ZCWPW1*/*PILRA*, *PTK2B*, *CELF1*/*SPI1*, *SORL1*, *FERMT2*, *SLC24A4/RIN3*, and *CASS4.* In this study, one earlier reported locus (*CD33*) was not found to be GWS, but a later study confirmed this being a genuine locus (discussed below). The potential of *PTK2B* being an AD locus was also reported in an earlier GWAS with a small sample size (*n* = 2229) in which multiple SNPs with *p* < 4E − 03 were reported in this gene [66]. A rare GWS variant in *TREM2* (p.Arg47His/rs75932628) was also discovered in 2013 [67] and subsequently replicated in independent studies [68]. In 2014, a follow-up meta-analysis of the summary statistics of suggestive IGAP loci with a Spanish sample of 4372 subjects (1808 case, 2564 controls) resulted in the identification of a novel GWS signal with a rare intronic *TRIP4* variant [69]. The IGAP data was further used in 2017 to identify rare coding variants in a 3-stage study using a whole-exome microarray in 34,174 samples in stage 1 followed by genotyping 35,962 independent samples in stage 2 and 14,997 samples in stage 3, totaling 85,133 subjects (37,022 cases, 48,402 controls) [70]. This study confirmed an earlier reported *TREM2*/p.Arg47His variant and identified another GWS *TREM2* variant (p.Arg63His) whose effect was independent from p.Arg47His. This study also reported GWS rare missense coding variants in novel *PLCG2* (p.Pro522Arg) and *ABI3* (p.Ser209Phe) genes.

In addition to the standard case–control genetic association study in GWAS that includes a set of subjects with a defined disease (cases) and a set of subjects who do not have a disease (controls), an alternative GWAS approach is a proxy case–control genetic association study in which relatives of affected (proxy cases) and un-affected (proxy controls) individuals are identified from a population-based cohort study. This approach is called as a genome-wide association study by proxy (GWAX) [71]. Although GWAX has lower power to detect association using proxy cases, it increases the sample size substantially and logistically it is more feasible than collecting standard cases. This approach has shown to be a valid approximation of future disease status for 12 common diseases, including AD, by showing replication of established genetic associations in GWAX. During 2017–2019, three AD GWAX were published using the UK Biobank (UKB) cohort, which were meta-analyzed with data from standard GWAS [71–73]. The first GWAX done in 2017 [71] used a subset of the UKB cohort comprising 14,482 proxy cases and 100,082 proxy controls (total 114,564 subjects) and identified APOE*4/rs429358 as the only GWS signal (*p* = 9.7E − 195). Next, GWAX data was meta-analyzed with the IGAP-2013 discovery GWAS data (*n* = 74,046), making the combined sample of 188,610 subjects (40,062 cases, 148,548 controls), which resulted in the identification of 4 novel loci: *HBEGF*/*APBB3*, *ECHDC3*, *SPPL2A*, and *SCIMP*/*RABEP1*. Interestingly, although all 4 loci were suggestively significant in the IGAP data (*p* range = 5.01E − 07 to 1.63E − 07), they were only nominal significant in the GWAX data (*p* range = 0.04 to 0.00026 to). Of the 26 top GWS SNPs, including APOE*4, 9 did not even achieve nominal significance in the GWAX data (*p* range = 0.46 to 0.059), indicating low power with proxy cases. Furthermore, *HBEGF* and *ECHDC3* loci were also identified in a transethnic GWAS published in 2017 (discussed below). The second GWAX/GWAS published in 2018 [72] included 314,278 individuals (27,696 maternal proxy cases, 14,338 paternal proxy cases) from the UKB cohort, also including subjects from GWAX-2017. GWAX was performed separately for maternal and paternal AD due to a 1.7-fold difference in disease prevalence — 9.6% and 5.5%, respectively. Six known GWS loci were identified in the UKB paternal and maternal GWAS meta-analysis. Then, UKB parental summary statistics were meta-analyzed with the IGAP-2013 discovery GWAS data (combined sample of 388,324 subjects, including 67,614 cases and 320,710 controls), which resulted in the identification 27 loci including 4 new ones: *ADAM10*, *KAT8*, *IL34*, and *ACE*. The third GWAX/GWAS published in 2019 [73] further expanded the sample to 455,258 subjects (71,880 cases, 383,378 controls) by meta-analysis in a 3-stage study. Stage 1 involved meta-analysis of clinically diagnosed 79,145 subjects (24,087 cases, 55,058 controls) derived from IGAP-2013 (*n* = 54,162), Alzheimer’s disease working group of the Psychiatric Genomics Consortium (PGC-ALZ; *n* = 17,477), and whole-exome sequencing (WES) data from the Alzheimer’s Disease Sequencing Project (ADSP; *n* = 7,506), which identified 18 known GWS loci. Stage 2 performed GWAX analysis on 376,133 individuals (47,793 proxy cases, 328,320 proxy controls) and identified 13 GWS loci, 8 of which overlapped with stage 1 and the lead SNPs of 3 other loci were not available in the stage 1 sample, including one in *TREM2*; one was in an earlier GWAX/GWAS-2018 [72]. Due to a strong genetic correlation (0.81) observed between clinically assessed AD status and AD-by-proxy as well as high concordance in the individual SNP effects in stage 1 and 2 analyses, meta-analysis of stage 1 and 2 results was conducted in stage 3 on the combined sample of 455,258. The combined meta-analysis identified 29 distinct loci, of which 7 were new: *ADAMTS4*, *HESX1*, *CLNK*, *CNTNAP2*, *APH1B*, *ALPK2*, and *AC074212.3*. The lead SNPs at 7 of the novel 9 loci were further tested in an independent Icelandic cohort (deCODE), including 6593 AD cases and 174,289 controls and only 5 of them were replicated at nominal significance. Four loci identified in IGAP-2013 (*MEF2C*, *NME8*, *CELF1*, and *FERMT2*) were not found to be GWS in this analysis, mostly due to the lower association signals in the UKB data set. However, the *CD33* locus, which was not GWS in IGAP-2013, was significant in the combined meta-analysis. *MEF2C* was also found to be GWS in the 2017 GWAX/GWAS [71].

In 2019, IGAP also expanded its stage 1 sample to 63,926 and along with stage 2 (*n* = 18,845) and stage 3 (*n* = 11,666) samples performed a meta-analysis on clinically assessed sample of 94,437 individuals (35,272 cases and 59,163 controls) [74]. This paper was published back-to-back with the GWAX/GWAS-2019 paper [73] and identified 25 GWS loci, including 3 new ones: *IQCK*, *WWOX*, and *ADAMTS1* [74]. IGAP-2019 also identified GWS *ADAM10* and *ACE* loci, which overlapped with GWAX/GWAS findings [72, 73]. It is noteworthy that the larger GWAX/GWAS-2019 dataset [73] showed no or only modest evidence of association for *IQCK* (*p* = 0.14), *ADAMTS1* (*p* = 0.016), and *WWOX* (*p* = 0.77). Two loci that were GWS in IGAP-2013 did not achieve GWS threshold in IGAP-2019 (*NME8*: *p* = 2.7E − 07 and *MEF2C*: *p* = 9.1E − 08).

More recently, two new GWAX/GWAS have been published using the largest discovery sample for family history of AD to date in the UKB. The first study included 53,042 unique individuals who were either diagnosed with AD or who reported a parent or sibling having dementia, and 355,900 controls, totaling 408,942 subjects [75]. This analysis identified 13 GWS loci, of which 3 were new (*NCK2*, *PRL*, and *FAM135B*). Next, GWAX results were meta-analyzed with the IGAP-2019 GWAS stage 1 sample of 63,926, resulting in a total sample of 472,868 subjects (75,024 cases and 397,844 controls). This identified 34 AD risk loci, including 4 novel loci near *SPRED2*, *NCK2*, *CCDC6*, and *TSPAN14*. Two additional novel loci achieved subthreshold GWS: *TMEM163* on chromosome 2 (*p* = 5.24E − 08) and *IKZF1* on chromosome 7 (*p* = 7.68E − 08). The *PRL* and *FAM135B* loci identified in the GWAX discovery sample showed no evidence of association in the IGAP-2019 stage 1 sample. Replication of the identified 34 loci was further sought in two data sets, including 7409 subjects from the GR@ACE study (4120 cases and 3289 controls) and 135,638 subjects from the FinnGen Biobank v.3 (3697 cases and 131,941 controls). Only about half of the 34 loci were nominally replicated (*p* < 0.05) in either of the two replication samples. Subsequently, meta-analysis was performed with all four datasets comprising a combined sample of 615,915 (82,841 cases, 533,074 controls), in which support for 3 of the 4 new loci (*NCK2*, *CCDC6* and *TSPAN14*) was increased, but weakened for *SPRED2* (*p* = 1.3E − 07). The second GWAX/GWAS expanded the study subjects to 1,126,563 that included 90,338 (46,613 proxy) AD cases and 1,036,225 (318,246 proxy) controls and reported 38 loci [76], including 7 novel loci ((*AGRN*, *TNIP1*, *HAVCR2*, *TMEM106B*, *GRN*, *NTN5*, *LILRB2*). Two of the novel AD loci (*TMEM106B*, *GRN*) are also known frontotemporal dementia (FTD) genes [77, 78], suggesting their potential roles in protein clearance rather than in specific disease-related protein aggregates [76]. In a recent paper available in preprint [79] using the largest number of 111,326 (46,828 proxy) AD cases along with 677,633 controls have identified the most number of 68 loci in one study that also included 35 new loci: *SORT1*, *ADAM17*, *PRKD3*, *WDR12*, *MME*, *IDUA*, *RHOH*, *ANKH***,** *COX7C*, *RASGEF1C*, *HS3ST5*, *UMAD1*, *ICA1*, *JAZF1*, *SEC61G*, *CTSB*, *SHARPIN*, *ABCA1*, *ANK3*, *BLNK*, *PLEKHA1*, *TPCN1*, *IGH* cluster, *SNX1*, *CTSH*, *DOC2A*, *MAF*, *FOXF1*, *PRDM7*, *WDR81*, *MYO15A*, *KLF16*, *SIGLEC11*, *RBCK1*, and *SLC2A4RG.* Another paper in preprint [80] has identified two of these loci (*SHARPIN*, *ATF*/*SIGLEC11*) in a total sample of 80,685 AD cases and 243,682 controls.

### *APOE*-Stratified GWAS

APOE*4 is the greatest risk factor for AD and is also the major contributor to the observed genetic variance. It is possible that in the presence of APOE*4, the effect of some significant genes may not be apparent or additional genetic factors may act in concert with APOE*4 to increase AD risk. In order to address this question, the first *APOE*-stratified GWAS-based on the presence (*E4* carriers) or absence (non-*E4* carriers) of APOE*4 was carried out in 2016 [81] on the IGAP-2013 sample comprising 19,559 APOE*4 carriers (10,352 cases, 9207 controls) and 34,152 non-APOE*4 carriers (7184 cases, 26,968 controls). Suggestive associations at *p* < 1E − 04 were examined in an independent sample comprising 1786 E*4 carriers (1250 cases, 536 controls) and 2417 non-E*4 carriers (718 cases, 1699 controls). A GWS signal was observed among non-APOE*4 carriers on chromosome 17 between *KANSL1* and *LRRC37A*, which is about 200 kb downstream from the *MAPT* gene that codes for the microtubule-associated protein tau found in AD neurofibrillary tangles. However, conditional analysis excluded the involvement of the *MAPT* gene or another gene distal to *LRRC37A*. Two subsequent WES studies using the overlapping ADSP dataset nominated *NSF* as a candidate gene in this region, which is located downstream from the *LRRC37A* gene [82, 83]. *NSF*/rs199533 is a coding variant (p.Lys702Lys), which was found to be the most significant *cis*-eQTL for *KANSL1-AS1* and *LRRC37A* in different brain regions and blood [83]. The *APOE*-stratified analysis also revealed that the previously established associations of *MSA4* and *TREM2* are mainly derived from non-APOE*4 carriers.

Altogether, 90 new loci were identified in addition to *APOE*, among White or European populations during the period from 2009 to early 2022 in at least one GWAS (Table 2). Although the combined sample size in AD GWAS and/or GWAX/GWAS seems large, there is considerable overlap in samples used in these studies and thus technically these studies are not independent.

### GWAS in Non-Whites

Although more older Whites are living with AD, older Black/African Americans and Hispanics are disproportionally more likely than older Whites to have AD [2]. Despite this fact, these population groups along with other non-White populations are understudied with regards to the genetic susceptibility to AD. The first large GWAS on African Americans age 60 and above was done in 2013 under the banner of ADGC, which comprised 5896 subjects (1968 case, 3928 control) [84]. In addition to *APOE*, the *ABCA7* locus was found to be GWS. Interestingly, although APOE*4 allele in an African genetic background confers lower risk than that observed for Whites, the effect size of *ABCA7* was double in African Americans (OR = 2.3) than reported previously in Whites. An expanded ADGC African American cohort, the largest to date, including 8006 subjects (2784 cases and 5222 controls), has been published recently [85], in which *APOE* was the only GWS locus and it replicated *ABCA7*/rs115550680 and 5q35/rs145848414 variants at *p* < 5E − 06 from the 2013-GWAS. Five additional GWS loci observed in Whites (*BIN1*, *TREM2*, *CD2AP*, *FERMT2*, and *WWOX*) were also replicated with nominal significance.

After adjusting for the effect of *APOE*, a novel GWS intergenic locus associated with rare variants was observed in African Americans on chromosome 15q26 near *ARRDC4* and *IGF1R* (Table 2). Another locus associated with rare variants on chromosome 11 was subthreshold GWS (*API5*/rs569584007; MAF = 0.01; *p* = 8.8E − 08). Five additional novel loci with rare variants (*SIPA1L2*, *WDR70*, *ACER3*, *PIK3C2G*, *RBFOX1)* and 4 with common variants (*EDEM1*, *ALCAM*, *GPC6*, *VRK3*) were observed at *p* < 1E − 06.

The previously reported AD-associated rare *COBL*, *AKAP9*, and *TREM2* variants in African Americans [86–89] were replicated in this larger GWAS as follows: *COBL*/rs112404845 (200 kb upstream of *COBL*) at *p* = 5.4E − 06, *AKAP9*/rs149979685 (p.Ser3771Leu) at *p* = 0.005, *AKAP9*/rs914662445 (p.Ile2558Met) at *p* = 0.01, *TREM2/*rs2234256 (p.Leu211Pro) at *p* = 0.001, *TREM2*/rs2234258 (p.Trp191X) at *p* = 1.4E − 03, and *TREM2/*rs7748513 at *p* = 3.6E − 05. The latter *TREM2* variant is 1 kb downstream of and in LD with the strongest associated *TREM2*/rs75932628 (p.Arg47His) variant in Whites, which is present sporadically in African Americans (MAF = 0.0009) [88], perhaps due to white admixture. The rare *COBL* and *AKAP9* variants are unique to people with African ancestry. Despite the fact that this was the largest GWAS in African Americans, its sample size compared with White GWAS is extremely small and thus only one GWS and some suggestive novel loci were identified.

A Chinese study that included 447 AD cases and 442 cognitively normal controls and 1745 mixed controls from the CONVERGE (China Oxford and Virginia Commonwealth University Experimental Research on Genetic Epidemiology) found *APOE* as the only GWS locus [90]. This study also tested 16 of the 21 IGAP-2013 GWS variants and found only 3 of them (*BIN1*, *CD2AP*, *FERMT2*) to be nominal significant in Chinese. An earlier study replicated *PICALM*, *BIN1*, *CLU*, and *MS4A4E* variants in Chinese [91]. Although known missense risk variants in *TREM2* have not been replicated in Chinese, a rare *TREM2*/rs2234255 coding variant in exon 3 (p.His157Try) has shown to confer a considerable risk for AD in Chinese [92].

Recently, a large two-stage GWAS conducted in Japanese comprising 5178 cases and 6520 controls identified a novel *SCARB2*/*FAM47E* locus on chromosome 4 in addition to the known *APOE* and *SORL1* loci [93]. The lead SNP and its proxies were eQTLs for both *FAM47E* and *SCARB2* genes in several brain tissues. Previously, the *SCARB2*/*FAM47E* locus has also been implicated with Parkinson disease (PD) [94]. *SCARB2* seems to be the candidate gene for both AD and PD as it plays a role in neuroinflammation and in the degradation process of α-synuclein [95].

### Transethnic GWAS

The transethnic meta-analysis approach that gathers results from multiethnic participants in existing studies has been found to be useful in discovering new loci for genetic traits and diseases [96–99]. This approach has enabled the identification of multiple associations previously unreported in European-only analyses [99]. In an effort to discover new AD loci, a two-stage transethnic GWAS was conducted in 2017 [100]. The stage 1 sample comprised 33,263 subjects, including 26,320 American Whites, 4983 African Americans, 1845 Japanese, and 115 Israeli Arabs as part of the ADGC. Stage 2 sample consisted of 26,287 White subjects (5813 cases, 20,474 controls) from IGAP-2013 after excluding the ADGC datasets. GWAS meta-analysis from both stages identified 3 GWS loci near *PFDN1/HBEGF* on chromosome 5, *USP6NL/ECHDC3* on chromosome 10, and *TSPOAP1* (formally *BZRAP1*)/*TSPOAP1-ASI* on chromosome 17. A fourth locus was identified based on a GWS interaction between APOE*4 and *NFIC*/rs9749589 SNP on chromosome 19 (Table 2) in which this SNP was protective in APOE*4 carriers (OR = 0.86; 95% CI: 0.80–0.83; *p* = 5.5E − 05), but with slight increased risk in non-APOE*4 carriers (OR = 1.12; 95% CI: 1.05–1.18; *p* = 1.2E − 03). Further evaluation of six GWS intergenic SNPs between *USP6NL* and *ECHDC3* genes revealed that these associations were exclusive to non-APOE*4 subjects (leading SNP: rs7920721, *p* = 2.7E − 09) [100]. As described above, two of the 4 loci identified in the transethnic GWAS (*PFDN1/HBEGF*, *USP6NL/ECHDC3)* produced suggestive evidence of association (*p* > E − 07) in IGAP-2013 and then became GWS after combining the IGAP-2013 data with 2017 GWAX. Thus, in 2017, these two loci were identified independently by this transethnic GWAS as well as GWAX/GWAS using the IGAP-2013 data. It is noteworthy that although the discovery sample in the transethnic GWAS was much smaller than used in the recent large Whites GWAS and GWAX, it still enabled the identification of novel loci. This highlights the value of combining data from diverse population groups in which diversity can be used to increase power for gene discovery [101]. Considering that *PFDN1/HBEGF* and *USP6NL/ECHDC3* loci were also identified in GWAX/GWAS-2017, *TSPOAP1*/*TSPOAP1-ASI* and *NFIC* loci can exclusively be attributed to the transethnic GWAS. Interestingly, the transethnic *TSPOAP1*/*TSPOAP1-ASI*/rs2632516 SNP was also GWS (*p* = 1.4E − 09) in the large case–control phase 1 sample of > 79 k, but it was less significant in the phase 2 proxy sample (*p* = 0.005) that yielded a meta-*p* of 9.7E − 07 [73]. However, another close by SNP in this region, rs2526380, was GWS in the meta-analysis (*p* = 2.6E − 08) in the 2019-GWAX/GWAS [73]. Thus, *TSPOAP1*/*TSPOAP1-ASI* locus was technically GWS significant in 2019-GWAX/GWAS, but it was not highlighted in the paper as such. Due to this reason, both the transethnic and GWAX/GWAS SNPs are included in Table 2.

A recent transethnic GWAS meta-analysis, comprising Japanese (5178 cases and 6520 controls) and Whites from the 2019 IGAP stage 1 data (21,982 AD cases and 41,944 controls), identified a novel locus, *OR2B2*, on chromosome 6 [93]. Future transethnic GWAS including larger non-white populations will help to identify additional AD novel loci.

In summary, GWAS and GWAX/GWAS have identified 95 loci for LOAD, which were GWS in at least one GWAS (Table 2). Of the 95 loci, one is unique to African Americans (*IGF1R*), one is unique to Japanese (*SCARB2*/*FAM47E*), and two were discovered using the transethnic GWAS approach (*NFIC*, *OR2B2*). Of the remaining 91 loci in Whites, one was discovered in the *APOE*-stratified analysis (*KANSL1*). Although the rare coding *COBL* and *AKAP9* variants observed in African American GWAS were not GWS, it seems that the *COBL* and *AKAP9* are genuine candidate genes for LOAD in people with African ancestry.

## Sequencing Strategy to Identify Additional AD Variants/Genes

As discussed above, substantial progress has been made via large-scale GWAS as well as meta-analyses of GWAS that have identified about 95 susceptibility loci for LOAD. However, GWAS-implicated genes/variants explain only a portion of the AD genetic variance [9–11] and much of the genetic variance remains unexplained. GWAS arrays that usually rely on LD to detect association signals fail to detect phenotypic association with functional or causal variants that are neither genotyped nor in LD with SNPs included in GWAS arrays even using imputation methods [102–104]. It is estimated that about 1% of the SNPs in the HapMap, which number in tens of thousands, are not in LD with other SNPs, and thus, they must directly be genotyped or sequenced for association analyses [105, 106]. To identify such variants as well as novel genetic variation affecting AD risk, the Alzheimer’s Disease Sequencing Project (ADSP) was implemented by the National Institute on Aging (NIA), and the initial phase included WES on about 11,000 AD cases and controls [103, 107]. The latest WGS data released from the ADSP in March 2021, and available to qualified investigator, was on about 15,000 AD cases and controls (https://dss.niagads.org/niagads-dss-releases-additional-17k-whole-genomes/) and the plan is to have WGS data from ~ 70,000 ethnically diverse and global cohorts in late 2022.

This first large-scale WES from ADSP [103] confirmed associations with common and rare variants in multiple previously established AD genes, including *APOE*, *ABCA7*, *HLA-DPA1*, *MS4A6A*, *PILRA*, *SORL1,* and *TREM2*. This study also identified three novel genes: one rare exome-wide significant variant in a long non-coding RNA gene, *AC099552.4* (*p* = 1.2E − 07) on chromosome 7; one common nearly exome-wide significant variant in *IGHG3* (*p* = 9.8E − 07) on chromosome 14; and a gene-wide significant association with *ZNF655*, including 9 high-impact rare variants (*p* = 5E − 06) on chromosome 7. A follow-up *APOE*-stratified WES analysis of ADSP and additional replication cohorts identified a near GWS association with a rare *GPAA1* exonic variant, rs138412600, on chromosome 8 (*p* = 7.81E − 08) among subjects lacking APOE*4. On the other hand, gene-based test of rare variants identified *IGHV3-7* on chromosome 14 (*p* = 9.75E − 16) and *SLC24A3* on chromosome 20 (*p* = 2.67E − 12) as possible novel genes in the ADSP discovery sample among APOE*4 carriers [82]. As mentioned above, a recent meta-GWAS analysis has identified GWS signals in the IGH (immunoglobulin heavy chain) gene cluster on chromosome 14 ([79]; Table 2), which confirms the WES findings about the involvement of *IGHG3* and *IGHV3-7* genes in AD risk. Human IGH seems to have inherent anti-amyloidogenic activity [108] and thus have a potential role in AD pathogenesis.

About 98% of the genome is non-coding and GWAS data indicate that > 90% of the disease and trait-associated variants of small effect sizes are non-coding, and many of them are concentrated in regulatory regions [109, 110]. Thus, WGS will identify additional variation in non-coding regulatory regions that will contribute to novel risk gene discovery, especially ultra-rare variants and copy number variants with large effect sizes than seen in GWAS, and will provide new molecular mechanistic insights for AD. Overall, the WGS approach offers several advantages over WES [111]: i) WGS covers both coding and non-coding regions, including the non-coding regulatory regions where GWAS have identified most of the AD-associated variants, ii) WGS even at 30 × coverage has shown to be more powerful than WES for detecting coding variants, and iii) WGS provides greater uniformity of sequencing reads, determination of insertions/deletions and copy number variations, and better cost per base than WES.

An early attempt to test the utility of WGS in AD has already begun. An association study of WGS conducted on 889 Han Chinese cases and controls along with 1745 mixed controls identified two suggestive loci (*GCH1*/rs72713460, *p* = 4.4E − 05 and *KCNJ15/*rs928771, *p* = 3.6E − 06) [90]. The lead SNPs in suggestive loci were not significant in the IGAP-2013 discovery data, indicating their potential ethnic-specific associations. A recent large WGS study was carried out in a family-based sample comprising 2247 subjects from 605 multiplex AD families followed by replication in an unrelated case–control sample of 1669 (983 cases) derived from the ADSP [112]. Although no GWS association was observed with rare sequence variants (MAF ≤ 1%), nominal meta-*p* were reported for 13 potential novel candidate loci, including 4 from single-variant analysis (*FNBP1L*, *SEL1L*, *LINC00298*, *C15ORF41*; *p* range = 1.1E − 02 to 2.4E − 04) and 9 from spatial-clustering (gene-based) analysis (*PRKCH*, *C2CD3*, *KIF2A*, *APC*, *LHX9*, *NALCN*, *CTNNA2*, *SYTL3*, *CLSTN2*; *p* range = 1.8E − 04 to 8.2E − 06). This clearly indicates that a much larger WGS sample is needed to discover novel GWS rare variants, and the availability of larger WGS data through the ADSP in 2022 may help to identify several new AD loci.

## Molecular Pathways

Alzheimer’s disease is a complex and heterogeneous disease where multiple biological pathways and their interactions appear to converge to its pathobiology. Characterization of AD-related gene network connectivity in a given pathway and its regulation and association to AD may help to provide new insights about the underlying biological mechanisms and eventually the identification of drug targets. Gene expression profiling in autopsied brain tissues from AD cases and controls has implicated distinct cell types and biological pathways in AD pathogenesis. These studies highlight the key role of microglia as well as subpopulation of oligodendrocyte and astrocyte cells and the immune and microglial biological pathways in AD [113–115]. GWAS provides a genetic approach to identify molecular pathways. In addition to the Aβ pathway, new genes identified in early GWAS suggested the involvement of immunity, lipid, and endocytosis pathways [116]. Data from recent larger GWAS in conjunction with functional genomics work also implicate the role of microglia, immune system, and protein catabolism of plaques as relevant to LOAD [76, 117, 118]. In contrast to psychiatric disorders and behavioral traits where GWAS variants are primarily present within neuronal enhancers and promoters, AD-associated variants are largely confined to microglial enhancers [117]. Protein–protein interaction (PPI) network for microglia associated AD-risk genes has been found to be highly connected and centered around ApoE, which contrasts to smaller in scope PPI networks observed for neurons and oligodendrocytes associated AD-risk genes. Whereas the microglia AD-associated genes highlight the gene ontology terms for immune function, the gene ontology terms for Aβ processing were associated with neurons, microglia, and oligodendrocytes [117].

## Moving from Genomics to Functional Genomics

Genomic localization of AD susceptibility loci is only the first step toward delineating the functional genomics of AD. The next important step is to identify the functional gene or genes among the many genes located in a given locus so that their involvement in AD pathogenesis could be further investigated. The exonic location of GWAS lead or sentinel SNPs might be considered causal because they can affect protein structure and function by altering amino acid sequence (non-synonymous variants) or can affect translation or protein stability, if located in 3′UTR, and transcription binding, if located in 5′UTR. However, > 90% of the GWAS-implicated variants are non-coding with no direct effect on protein structure or function and many are located far away from the closest known gene. Generally, the gene located closest to the GWAS lead SNP in a region is considered functional, which often is not the case [119].

A general framework to identify the functional gene or genes in a given locus has been proposed [120] that involves the use of conditional analyses to determine if there is a single or multiple independent signals within a locus followed by integration of disease-associated SNPs with publically available multiomic datasets. Many non-coding GWAS-associated SNPs are shown to be eQTLs (expression quantitative trait loci), suggesting they could act through altering gene expression. Other non-coding variants may also affect disease risk by altering DNA methylation (mQTLs), DNase hypersensitivity (dsQTLs), TF binding (bQTLs), or protein levels (pQTLs). Several studies have shown that GWAS-associated non-coding variants are enriched in predicted transcriptional regulatory regions, known as “*cis*-regulatory elements,” and they appear to affect disease risk by modifying the function of regulatory elements in disease-relevant cell types, with subsequent changes in target gene expression. For example, autoimmune disease–associated variants were mapped to promoters and enhancers active in B and T cells, neurological disease risk variants were mapped to promoters and enhancers active in brain tissues, and fasting blood glucose-associated variants were mapped to regulatory elements in pancreatic islets [110]. As mentioned above, AD-associated variants are largely confined to microglial enhancers followed by other brain cells [76, 113–115, 117].

As shown in Table 2, sixteen GWAS lead SNPs may directly be functional due to their locations either in coding exons (*APOE*, *SORT1*, *MME*, *TREM2*, *PILRA*, *APH1B*, *PLCG2*, *ABI3*, *IL34*) or in 3′UTR (*ADAMTS4*, *WDR12*, *CTSB*, *KLF16*) and 5′UTR (*RHOH*, *WDR81*, *CD33*). In addition to the lead coding SNPs in the above 9 genes, GWS non-synonymous variants have also been found in the *C1R*, *PVRIG* (*PILRA* locus), *MS4A6A*, *ACE*, and *CD33* and multiple genes in the *APOE* locus, including *BCL3*, *CBLC*, *BCAM*, *TOMM40*, *APOC4-APOC2*, *NKPD1*, *EXOC3L2*, and *ENSG00000267114* [73–75]. Many of these genes harboring coding variants are strong candidate genes for AD. Multiple loss-of-function coding variants in *ABCA7* are associated with AD risk (*p* = 2.2E − 13), strongly suggesting its direct involvement in AD pathogenesis [121]. *TREM2*, *PLCG2*, and *ABI3* genes with rare coding variants are highly expressed in microglia and strongly support the involvement of microglia-mediated innate immune response in the etiology of AD. The *COBL* and *AKAP9* are also likely candidate genes for AD as rare coding risk variants discovered in these genes are unique to people with African ancestry.

Conditional analyses done on AD-associated variants in three recent large GWAS meta-analyses [73–75] identified at least two independent signals in 10 loci (*BIN1*, *PKT2B*/*CLU*, *ABCA7*, *NCK2*, *EPHA1*, *ADAM10*, *ACE*, *APP*/*ADAMTS1*, *TREM2*, and *APOE*), suggesting that there may be at least 10 additional loci which are not included in the currently known approximately 95 loci. Although *PKT2B* and *CLU* are two distinct loci on chromosome 8, there is some evidence to suggest that these two loci might physically interact to affect the AD risk through the same biological mechanism [75]. For this reason, some studies list them as one locus. However, considering that their tissue gene expression is not the same, probably they should be treated as two distinct loci until more experimental data is available. Of the two independent signals in the *APP*/*ADAMTS1* locus, one seems to be in the *APP* gene, in addition to the lead *ADAMTS1*/rs2830500 signal (*p* = 2.6E − 08) located about 51 kb upstream of *ADAMTS1*. The second most significant SNP in this locus is located in an *APP* intron (rs48170900; *p* = 1.0E − 07) [75], and its association is independent of *ADAMTS1*/rs2830500 (*D*′ = 0.0002; *R*^2^ = 0.0), suggesting that *APP* and *ADAMTS1* are two distinct loci on chromosome 21. Noteworthy, *APP*/rs48170900 was found to be GWS (*p* = 4.8E − 08) when a relaxed relatedness threshold was used for proxy AD cases and controls [72]. If *APP* is confirmed to be a distinct locus then this would provide further evidence of the involvement of common variants with AD risk in addition to the association of rare *APP*/rs63750847 (p.Ala673Thr) coding variant with AD. Interestingly, there were 8 GWS independent signals in the *APOE* locus, but they were not considered in association analyses due to the very strong effect of APOE*4 on AD risk [73]. A limitation of conditional analysis is that it can overlook functional variants it they are in strong LD with the lead SNP.

The four recent large studies also performed functional genomics analyses by employing multiple overlapping approaches to link the AD-associated variants with potential functional genes [73–76]. The IGAP-2019 study, which identified 25 loci [74], prioritized candidate genes using five strategies: i) annotation and gene-based testing for deleterious coding, loss-of-function, and splicing variants, ii) eQTL analyses, iii) evaluation of transcriptomic expression in AD clinical traits, iv) evaluation of transcriptomic expression in AD-relevant tissues, and v) gene cluster/pathway analyses. The outcome of these strategies provided strong support for the *APOE*, *ABCA7*, *BIN1*, *TREM2*, *SORL1*, *ADAM10*, *SPI1*, and *CR1* as the AD risk genes. The 2019 GWAX/GWAS that identified 29 loci [73] employed genome-wide gene-based association analysis using Multimarker Analysis of GenoMic Annotation (MAGMA) along with three gene-mapping strategies implemented in functional mapping and annotation (FUMA): positional gene mapping, eQTL gene mapping, and chromatin interaction mapping. All these four strategies implicated 16 functional genes, including *HLA-DRA*, *HLA-DRB1*, *PTK2B*, *CLU*, *MS4A3*, *SCIMP*, and *RABEP1* and 9 genes in the *APOE* locus (*IGSF23*, *PVR*, *BCAM*, *PVRL2*, *TOMM40*, *APOE*, *APOC1*, *APOC4*, and *CLPTM1*). Brain-specific eQTL and mQTLs provided further functional evidence in favor of *PVRL2*, *TOMM40*, and *APOC4*. Identification of multiple potential functional genes along with multiple independent signals unveiled in conditional analyses highlights the complex LD structure in the *APOE* locus.

Schwartzentruber et al. [75] performed functional genomics analyses on 37 loci, including 34 GWS and 3 suggestive loci (*IKZF1*, *TSPOAP1*, and *TMEM163* with *p* < 5E − 07). Comprehensive eQTL colocalization, annotation, fine-mapping, and network analyses were employed. Excluding *APOE*, eQTL colocalization identified 80 distinct genes at 27 loci that included, among others, *PTK2B*, *BIN1*, *PILRA*, *CD33*, *TREM2*, *FCER1G*, *TSPAN14*, *APH1B*, and *ACE*. Using three distinct fine-mapping methods, 21 variants with mean causal probability > 50% across the fine-mapping methods were identified. The top 13 GWS variants with causal probability > 50% included the following: *SPRED2*/rs268120 (56% probability), *NCK2*/rs143080277 (100% probability), *BIN1*/rs6733839 (100% probability), *INPP5D*/rs10933431 (83% probability), *PILRA/*rs1859788 (60% probability), *ECHDC3/*rs7920721 (64% probability), *SORL1/*rs11218343 (100% probability), *APH1B/*rs117618017 (90% probability), *PLCG2/*rs12444183 (69% probability), *ABCA7/*rs12151021 (71% probability), *CD33/*rs12459419 (66% probability), *CASS4/*rs6014724 (55% probability), and *ADAMTS1/*rs2830489 (72% probability). Of these potential causal variants, *BIN1*/rs6733839C > T, located 20 kb upstream of *BIN1*, creates a binding site for the MEF2C transcription factor that facilitates hippocampal-dependent learning and memory [122], and this SNP is an eQTL for *BIN1* in primary microglia and induced pluripotent stem cell-derived macrophages (IPSDMac) [123]. This suggests that this variant affects AD risk by increasing *BIN1* expression in microglia and IPSDMac due to the increased binding of MEFC2. Although *BIN1* and *MEF2C* are expressed in multiple tissues, their coexpression was found only in primary microglia and IPSDMac [123]. Another independent signal in *BIN1*, rs13025717, is an eQTL for *BIN1* in monocytes and together with rs6733839 has been implicated with AD risk through integration of single brain cell and peripheral myeloid epigenomics [124, 125]. As mentioned above, *APH1B/*rs117618017 (p.Thr27Ile) is the lead GWS variant in this locus and affects the APH1B protein structure, which is a component of γ-secretase. This structural change in APH1B may directly affect its function and the ensuing APP processing and AD risk. However, rs117618017 was also found to be an eQTL for *APH1B* in monocytes, neutrophils, and T cells in which the effect “T” allele was associated with higher *APH1B* expression, which may also explain its association with higher AD risk [75]. *PILRA*/rs1859788 (p.Gly78Arg) is also the lead GWS SNP in this locus. Although *CD33*/rs12459419 (p.Ala14Val) is not the lead SNP in this locus, it is GWS (*p* = 1.3E − 08) and is a strong splicing QTL, suggesting that it is likely the functional variant. Likewise, *CASS4/*rs6014724 is the lead GWS SNP in this locus and is an eQTL for *CASS4* in monocytes and whole blood. *CASS4/*rs17462136 is another GWS variant (*p* = 1.01E − 09), which is located in 5′UTR and is predicted to decrease transcription binding [75].

In addition to individual functional genomics analyses, Schwartzentruber et al. [75] also developed a comprehensive gene prioritization score based on quantitative information derived from five predictors: gene distance to lead SNPs, eQTL colocalization, network score, bulk and single-cell gene expression, and the sum of fine-mapped probability for any coding SNPs within a gene. To identify weights for the predictive features, two models were employed and then the average of the predictions from the two models was used as the final gene prioritization “model score or probability” to determine the most likely functional genes for AD across GWAS loci. There were 12 most confidently prioritized genes with a model score or probability of > 0.80: *PILRA*, *APH1B*, *PLCG2*, *SPI1*, *SORL1*, *CD33*, *CASS4*, *BIN1*, *CR1*, *ACE*, *ABCA7*, and *TSPAN14*; two additional genes, *PTK2B*, and *CD2AP*, had a model score of 0.79. With the exception of *CR1*, *CD2AP*, and *SPI1*, the other 11 genes overlap with genes identified from the eQTL colocalization and/or causal variants evidence. The latest and the largest published GWAX/GWAS in 2021 [76] performed functional genomics analyses on 38 loci and, based on the position and eQTL information from brain and immune tissues, identified 989 genes. Further colocalization analysis identified a candidate causal gene in 9 of the 38 loci, including *TNIP1*, *MADD*, *APH1B*, *GRN*, AC004687.2, *ACE*, *NTN5*, *CD33*, and *CASS4*.

The success of the integration of GWAS variants with omics data depends on the size of the tissue-specific omics data and the relevancy of the available tissues and cells to AD. Evidence suggests that regulatory causal variants have modest and cell-type specific effects [120], and this requires a well-powered QTL data to detect modest effects [126]. Microglia is one of the most relevant cell type to AD; however, currently, microglia QTL data is available on a small number of brain tissues [75, 124], leading the investigators to also use peripheral myeloid cells (monocytes and macrophages) in AD functional genomics studies. A recent such study has integrated AD GWAS with multiple peripheral myeloid genomic datasets [125], which nominated multiple candidate genes: *BIN1*, *SP1*, *ZYX*, *EPHA1*, *MS4A6A*, *MS4A4A*, *PILRA*, *RABEP1*, *SCIMP*, *PTK2B*, *GPR141*, *SPPL2A*, and *CD2AP*. Noteworthy, the *EPHA1/EPHA1-AS1* association signal is an eQTL for the *EPHA1-AS1* noncoding RNA both in primary microglia and in IPSDMac [123], suggesting that the AD risk may be mediated by *EPHA1-AS1* expression levels.

Although the functional genomics analyses in each study summarized above was not done on the same set of genes and not all candidate genes in a given locus were examined, for now, the following have the strongest evidence to be considered as the causal genes for further cell-based and/or animal model studies: *APOE*, *ABCA7*, *BIN1*, *TREM2*, *SORL1*, *SPI1*, *CR1*, *PTK2B*, *PILRA*, *CD2AP*, *APH1B*, *PLCG2*, *MS4A4A*, *MS4A6A*, *CD33*, *ADAM10*, and *ABI3*. Clearly, this is only a tentative list, which will certainly grow as functional genomics analyses are performed simultaneously on all known and GWS subthreshold genes on well-powered QTL data in AD-relevant tissues and cells.

## Concluding Remarks and Future Directions

Since 2009, the horizon of the AD genetics landscape has expanded enormously with the identification of at least 95 risk loci for LOAD, harboring both common and rare variants. With the projected availability of WGS data on large number of AD cases and controls in 2022, there is a high likelihood of discovering numerous addition rare variants in the existing and novel genes/loci, which certainly will lead to delineate the genetic architecture of AD. It is noteworthy, however, that the overall number of AD-associated loci remains low as compared with other neurodegenerative and psychiatric disorders [127–129]. Like most other common diseases and traits, LOAD is also considered as a polygenic disease with the possible contribution of thousands of variants with small effect to its overall heritable risk [130]. However, recent data suggest that LOAD may be less polygenic than psychiatric diseases, and traits related to intelligence, cognitive ability, and educational attainment [131] or even it has an oligogenic architecture requiring only ~ 100 common SNPs [132]. Another recent study predicted the number of causal SNPs for LOAD to be about 11,000 [133]. These predictions are far less than the estimates of causal SNPs for other complex diseases and traits (Table 3).

**Table 3:** Estimates of causal SNPs for complex diseases/traits^a^

Disease/trait	Number of casual SNPs
Alzheimer’s disease	99^b^
Alzheimer’s disease	11,200
Parkinson’s disease	34,000^b^
Major depression	173,000^b^
Schizophrenia	185,000^b^
Schizophrenia	582,000
Bipolar disorder	651,000
Crohn’s disease	10,500
Ulcerative colitis	12,700
Coronary artery disease	20,700
Total cholesterol	12,700
LDL-cholesterol	64,300
HDL-cholesterol	27,900
Intelligence	140,000
Education	158,000
Body mass index	25,700

^a^Adapted from Holland et al. 2021 [133]

^b^From Zhang et al. 2020 [132]

Of the 58–70% reported heritability of LOAD [5, 6], the common SNP-based heritability from earlier known LOAD loci has been estimated to be up to ~ 35% [9–11, 131, 132], which needs to be re-evaluated based on the latest and larger identified loci. Some of the missing heritability may also be discovered using alternative approaches to the case–control design. Studies focusing on AD-related quantitative endophenotypes and biomarkers that manifest earlier than clinical AD provide a powerful alternative approach to identify not only additional AD-related genes, but it may also help to uncover underlying mechanisms for AD and disease progression that cannot be obtained from case–control studies. Some successful examples of this approach have been demonstrated in GWAS on AD biomarkers and endophenotypes, including amyloid and tau [134–137], neurofilament light (NFL) [138, 139], neuropathologic features [140] resilience [141], psychosis [142], and AAO [132, 143–145], resulting in novel loci. Thus far, the focus of the vast majority of genomic studies in LOAD has been on European or European-derived White populations in overlapping subjects, despite the fact that the prevalence of AD is disproportionally higher in certain non-White groups. Special efforts need to be made to recruit and collect genetic material from a large number of non-White populations for meaningful and well-powered genetic association studies in order to identify potential novel ethnic-specific genetic factors, which is an essential step to complete the AD genetics landscape.

Even though the efforts to delineate the genetics landscape of AD continue, it is now time to divert attention and resources towards identifying the causal genes in all the identified loci so that the underlying disease causing biological mechanisms and pathways could be understood. A series of in silico functional genomics analyses have already begun to prioritize putative functional genes, but this effort has largely focused on study-specific-identified loci rather than on all the identified loci. Nevertheless, these studies have identified some promising candidate functional genes that will need to be tested further in cell-based systems and/or animal models to probe their roles in the pathogenesis of AD.

The ultimate goal of integrating genetic and functional findings is to discover novel pathways that may converge in the pathobiology of this heterogeneous disorder and eventually to identify drug targets for therapeutic treatment [146]. Due to the complexity of the underlying biological mechanisms and pathways causing the heterogeneity in the pathobiology of AD, a single drug target may not be therapeutic or it may work in only subset of patients. A combination drug treatment approach, as used for some other chronic diseases, has also been envisioned for AD [43]. In fact, anti-Aβ and anti-tau therapies have been recommended to be used simultaneously [147] because both Aβ and tau may act in parallel to exert their detrimental effects on AD [148, 149]. Despite the lack of clear success of anti-Aβ monotherapy for symptomatic AD in clinical trials, recently, the US Food and Drug Administration (FDA) has granted authorization to the monoclonal antibody aducanumab as part of its “accelerated approval” pathway (treatments that may reasonably likely, but not certain to help patients), which has met with skepticism as well as hope [150, 151] because this is the first new drug for AD since 2003. It is highly likely that functional genomics-guided discoveries would lead to the identification of novel druggable targets and eventually therapeutic treatment of this devastating disease.

## Supplementary Information

Below is the link to the electronic supplementary material.

Supplementary file1 (PDF 499 kb)
